# The effect of using an interactive booklet on childhood respiratory tract infections in consultations: Study protocol for a cluster randomised controlled trial in primary care

**DOI:** 10.1186/1471-2296-9-23

**Published:** 2008-04-24

**Authors:** Nick A Francis, Kerenza Hood, Sharon Simpson, Fiona Wood, Jacqueline Nuttall, Christopher C Butler

**Affiliations:** 1 South East Wales Trials Unit, Department of Primary Care and Public Health, School of Medicine, Cardiff University, Neuadd Meirionnydd, Heath Park, Cardiff, CF14 4XN, UK

## Abstract

**Background:**

Respiratory tract infections in children result in more primary care consultations than any other acute condition, and are the most common reason for prescribing antibiotics (which are largely unnecessary). About a fifth of children consult again for the same illness episode. Providing parents with written information on respiratory tract infections may result in a reduction in re-consultation rates and antibiotic prescribing for these illnesses. Asking clinicians to provide and discuss the information during the consultation may enhance effectiveness. This paper outlines the protocol for a study designed to evaluate the use of a booklet on respiratory tract infections in children within primary care consultations.

**Methods/Design:**

This will be a cluster randomised controlled trial. General practices will be randomised to provide parents consulting because their child has an acute respiratory tract infection with either an interactive booklet, or usual care. The booklet provides information on the expected duration of their child's illness, the likely benefits of various treatment options, signs and symptoms that should prompt re-consultation, and symptomatic treatment advice. It has been designed for use within the consultation and aims to enhance communication through the use of specific prompts. Clinicians randomised to using the interactive booklet will receive online training in its use. Outcomes will be assessed via a telephone interview with the parent two weeks after first consulting. The primary outcome will be the proportion of children who re-consult for the same illness episode. Secondary outcomes include: antibiotic use, parental satisfaction and enablement, and illness costs. Consultation rates for respiratory tract infections for the subsequent year will be assessed by a review of practice notes.

**Discussion:**

Previous studies in adults and children have shown that educational interventions can result in reductions in re-consultation rates and use of antibiotics for respiratory tract infections. This will be the first study to determine whether providing parents with a booklet on respiratory tract infections in children, and discussing it with them during the consultation, reduces re-consultations and antibiotic use for the same illness without reducing satisfaction with care.

**Trial registration:**

Current Controlled Trials ISRCTN46104365

## Background

Acute respiratory tract infections (RTIs) are the most common illnesses experienced by individuals of all ages worldwide [1]. Children, who experience more illness episodes than any other age group, will on average have between five and six respiratory infections per year [1]. In the United Kingdom, 97% of pre-school age children will consult with a doctor at some point, mostly for symptoms related to respiratory tract infections [2]. These illnesses are the most common reason for patients to consult in primary care, and children consult more than any other age group [3]. In addition, around one in five children consulting because of a RTI will re-consult for the same illness episode [4], a proportion that has changed little over the past thirty years [5]. Parents frequently describe anxiety and disempowerment when coping with respiratory tract infections in their children [6,7]. These problems may be addressed by the provision of clear, reliable information [7,8].

Furthermore, most respiratory tract infections benefit very little from treatment with antibiotics [9-13], yet their use continues to be widespread [14,15] with children receiving more antibiotics than any other age group [16]. Approximately 25% of consultations with children with a RTI result in an antibiotic prescription [16], and approximately half of all children aged 0 – 4 will receive an antibiotic prescription in any one year period [17]. Unnecessary antibiotic prescribing results in wasted healthcare resources, leads to a cycle that encourages further consulting in the future for similar illnesses [18] and contributes to the problem of antibiotic resistance [19].

Parental factors influencing the decision to consult include concerns, knowledge, beliefs, and expectations. Parents often fear serious illness, and worry that they will not be able to recognise symptoms of serious illness [6]. Despite their concerns, a number of parents worry about 'bothering' their general practitioner with these illnesses [20,21]. They also lack knowledge about the likely risks and benefits from antibiotic treatment, and the normal duration of illness [22]. Patients may hold beliefs about the causes of respiratory tract infections [22-24], the meaning of symptoms [25,26], and the effectiveness of medications [22,23,25,27], that are at odds with biomedicine.

Providing parents with written information about respiratory tract infections in children may help alleviate anxiety and improve parental feelings of satisfaction and enablement. In adults, the use of leaflets describing the expected duration of symptoms and giving advice about self-help for lower respiratory tract infections, has resulted in reductions in re-consultations [28] and antibiotic prescribing [29].

Expectations that patients (and parents) bring to consultations can have an impact on outcomes. There is an increased likelihood that antibiotics will be prescribed when parents consult with expectations for an antibiotic prescription [30]. However, clinicians seldom explicitly enquire about expectations [31], and often over-estimate the expectation for antibiotics [21]. A clinician's perception of an expectation for antibiotics is associated with an even greater likelihood of prescribing than actual patient expectations [30,32]. Parents value a thorough examination, explanation, reassurance and advice or guidance more than a prescription for antibiotics [21,33].

Other studies have evaluated the use of printed patient information on respiratory tract infections. Sending booklets on a range of minor illnesses (including respiratory tract infections) to patients' homes has been shown to have little impact on consultation rates in a number of studies [34-38]. However, an editorial accompanying the two most recent of these studies suggested that the use of written material to support the management of minor illnesses was more likely to be of value if it was used within the consultation and was context-specific [39].

A study conducted in the 1980's in one health centre in the United States showed a reduction in consultation rates for patients provided with a pack containing a pamphlet, sticker and thermometer [40]. However, this study was limited by non-random allocation, and post-allocation exclusion of subjects with chronic illnesses. In another US study, parents were randomised to receive educational materials on either the use of antibiotics or injury prevention [41]. There were no differences in consultation rates or antibiotic prescribing between these two groups, which may reflect the need to provide parents with positive information about managing an illnesses rather than a negative message about a treatment option (antibiotics). A more recent non-randomised US study examined the role of patient educational materials and providing clinicians with prescribing profiles and practice guidelines [42]. This led to reductions in antibiotic prescribing for bronchitis in adults, but not for paediatric pharyngitis.

Printed educational materials have frequently been used as part of larger multi-faceted interventions [43-48]. A number of these interventions have been associated with either a reduction in prescribing or improvements in parental knowledge and awareness about antibiotics. However, it is not possible to determine the role of the educational materials within these complex interventions, and in none of these studies were the materials designed for use specifically within the consultation.

We set out to address high levels of consulting and antibiotic prescribing, and parental disempowerment and dissatisfaction, by developing a booklet on respiratory tract infections in children, designed to be used within primary care consultations and then provided to parents as a take-home resource. The booklet and clinician training have theoretical roots in Social Cognitive Theory [49,50] and the Theory of Planned Behaviour [51,52]. The key aspects of these theories incorporated in the intervention are outcome and efficacy expectations. Behaviour change is more likely if the individual believes in the importance or value of change (outcome expectations) and feels that they have the confidence or skills to change (efficacy expectations). To enhance the likelihood that the intervention will result in reductions in health service utilisation and antibiotic prescribing it has been aimed at both clinicians and parents. Clinicians are provided with information about the implications of high levels of consulting and antibiotic use, in order to increase outcome expectations. They are also provided with specific tools to facilitate change (efficacy expectations). These tools include communications strategies provided within the training programme, and the study booklet, which acts as an aide memoir and a prompt to enhance communication within the consultation. Similarly, we aim to influence the behaviour of parents by providing them with information about the importance of change (benefits of self-management and implications of overuse of antibiotics), and by attempting to enhance their confidence and skills. We aim to achieve the latter through use of the study booklet which encourages them to have their concerns addressed within the consultation, and provides them with clear, relevant information about their child's illness.

### Main research questions

Our main aim is to determine whether the pragmatic use of this intervention can result in a reduction in the proportion of children who re-consult during the same illness episode. We will also examine the impact that use of this intervention has on: antibiotic prescribing and use, parental satisfaction, parental enablement, intention to consult with a similar illness in the future, illness costs, and consultation rates for RTIs over the following year. In this paper we describe the study protocol.

## Methods

**Figure 1:**
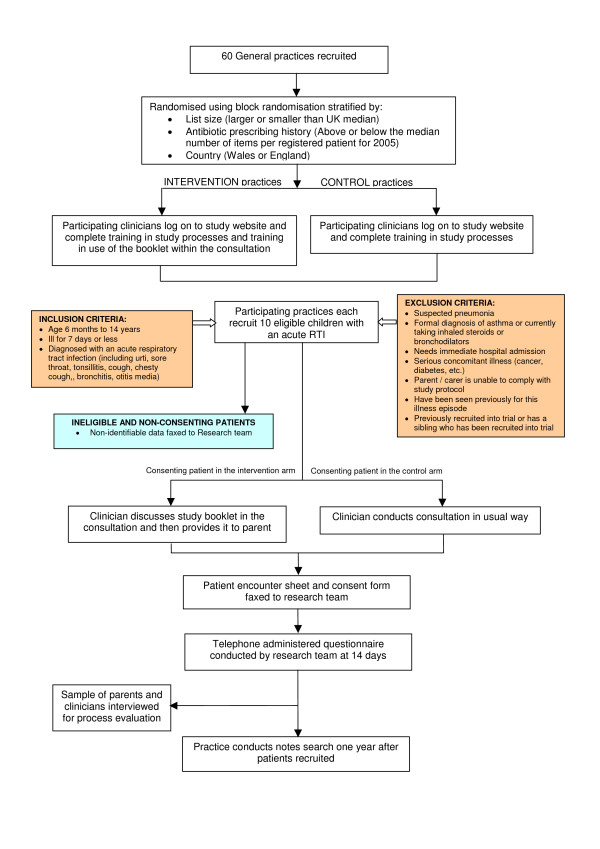
Study flow chart.

This will be a cluster randomised controlled trial with randomisation at the level of the general practice. Recruited general medical practices will be randomised to one of two arms; use of an interactive booklet or usual care (see Figure 1).

### The intervention

The study booklet was developed through a multi-stage process, which is outlined below and will be reported in full elsewhere.

The aims and broad content areas were decided through a number of 'brainstorming' sessions held by a multidisciplinary development group. Systematic searches were undertaken to identify existing patient information leaflets and literature on development of patient educational materials, management of respiratory tract infections in children, healthcare communication and shared decision-making. These materials were reviewed and synthesised, and a draft booklet (2 sides of A4 paper) was developed. This was presented to a number of parent focus groups, individual parents through an interview process, and two general practitioner focus groups. In addition, six practising academic GPs, two practising paediatricians, and a 'basic skills professional', who focused on improving readability, reviewed the emerging booklet. This process led to important changes to content and design. The design was also enhanced through input from a professional graphic designer. The end result was an eight-page A5 booklet, the content of which is summarised in Table 1.

**Table 1:** Summary of study booklet content

**Section name**	**Contents**
**Who is this booklet for?**	General introduction and advice on who the leaflet does not apply to (under 6 months, children with chronic illnesses)
**Prompts**	Prompts to remind the practitioner (and the parent) to discuss the parent's main concerns and their expectations.
**Fever**	Facts about fever and advice on managing it.
**Temperature fits (Febrile Seizures)**	General information and advice on management.
**Cough/Chesty Cough**	General information, graphical representation of normal duration, advice about management, and information about the effectiveness of antibiotics.
**Common Cold**	Information about frequency and graphical representation of normal duration. Advice on the effectiveness of antibiotics.
**Green phlegm/Snot**	Advice about the interpretation of discoloured nasal discharge.
**Sore Throat**	General advice, graphical representation of the normal duration, information about the effectiveness of antibiotics.
**Earache**	General advice, graphical representation of the normal duration, information about the effectiveness of antibiotics.
**Croup**	Information about symptoms and management (including signs of respiratory distress) and the effectiveness of antibiotics.
**Not Eating/Drinking**	Advice on these symptoms including signs of dehydration.
**What can I do?**	General section on management advice.
**Why not take antibiotics?**	Information about the potential disadvantages to use of antibiotics.
**When should I seek further help?**	Comprehensive section providing a description of symptoms and signs suggestive of serious illness, including pictures of a meningitis/septicaemia rash (and the 'tumbler test'). Also advice on other situations that should prompt re-consultation.
**Contact details**	Space for clinicians to write 'in-hours' and 'out-of-hours' contact numbers. Contact details for NHS direct (national health information service).

The aims of the interactive booklet are to act as an evidence-based information resource for parents, an aide memoir for clinicians, a tool to help set realistic expectations, and a prompt to enhance communication within the consultation. The booklet is described as 'interactive' because it has been designed for use within consultations to facilitate interaction between the clinician and the parent. It aims to achieve this by providing specific prompts which encourage discussion of the parent's main worries, and their expectations for the consultation. In addition, the booklet includes boxes and spaces, which allow for personalisation.

### Clinician training

All participating clinicians will be provided with training in study processes. This includes the background and aims of the study, how to recruit patients, inclusion and exclusion criteria, obtaining informed consent (including determining when a child should be asked to provide consent), and how to complete the patient encounter sheet (including the importance of accurate data collection). This training will be provided through a dedicated website, accessed by means of a username and password provided to all participating clinicians [53].

In addition to the training in study processes, clinicians in practices randomised to the intervention arm will complete a training module on use of the study intervention (booklet) within their consultations. Unique log-in details provided to these practitioners will automatically ensure that they are provided access to the additional training module. This training describes the contents of the booklet, encourages its use within the consultation, and encourages clinicians to use the booklet to facilitate the use of certain communication skills; namely exploring the parents' main concerns, asking about their expectations, and discussing prognosis, treatment options, and what should prompt re-consultation. The training incorporates videos that demonstrate use of the booklet in a consultation, as well as audio feeds, pictures, and links to study materials. This part of the training will take approximately 40 minutes to complete.

Clinician training will be monitored through the study website. This will allow the study team to identify whether a clinician has logged on to the site, how much time they spent on it, and which pages they have viewed. This will allow for a reasonable assessment of whether or not participating clinicians are completing the required training. Clinicians will be asked to complete all their required training before starting to recruit patients.

### Sample size estimation

The primary outcome is re-consultation for the same illness episode. In order to show a reduction in the proportion of children re-consulting from 20% to 10%, with 80% power, and a 5% significance level, we would require 438 participants for an individually randomised trial. From a previous study of upper respiratory tract infections in children [4], we calculated an intra-cluster coefficient of 0.04 for re-consultation rates. Using 60 clusters (practices) we would need 524 participants to have the same power as an individually randomised trial. To allow for loss to follow-up and missing data we have decided on a target recruitment of 600 participants, 10 from each practice.

### Recruitment of general practices

General practices will be recruited from throughout England and Wales. We will use a database of practices in Wales, and a number of UK Clinical Research Network primary care local research networks to contact practices in a wide range of regions. Recruited practices are asked to sign a Study Agreement. Practices can have one or more participating clinicians (general practitioners, nurse practitioners, or practice nurses), however each participating clinician must consult regularly with children presenting with acute illnesses. All clinicians in the same practice are allocated to the same trial arm.

### Randomisation

Recruited practices will be randomised using block randomisation stratified by list size, antibiotic prescribing rate, and country (Wales or England). Each recruited practice will provide a list size which is compared to the mean for England and Wales. Antibiotic prescribing rates (antibacterial items prescribed per 1000 registered patients for 2005) for each practice will be obtained and these will be compared with the mean rate for each country. For each of the eight strata the study statistician will create a randomisation table using random permuted block sizes. These tables will be kept securely and allocation for each practice will be provided only after the practice has agreed to participate and the practice ID and stratification variables are provided to the statistician.

### Patient recruitment

Participating clinicians are asked to invite sequential eligible patients (see Figure 1) consulting with a respiratory tract infection (including common cold, cough, bronchitis or chesty cough, sore throat, tonsillitis, and ear ache). Exclusions (see Figure 1) include children with asthma, pneumonia, and those with ongoing serious disease (cancer, kidney disease, heart disease, etc.) Parents will be asked to provide informed consent, and where a child is deemed capable of consenting, they will be asked to provide consent in addition to the parental consent.

### Data collection

In order to assess possible selection bias we will collect non-identifiable data on all 'potentially eligible patients'. This includes recruited patients as well as those approached but then deemed to be ineligible, those who decline participation, and those not recruited for other reasons (clinician was too busy, etc.) For these patients we will collect: date of consultation, duration of illness, age in years, gender, and presence or absence of the following symptoms: cough, earache, sore throat, runny nose, fever, looks unwell (subjective assessment by clinician). For eligible, consenting patients, we will also collect the patient's and parent's names, contact details (address and phone numbers), and the child's date of birth.

#### Follow up data collection two weeks after the initial consultation

Most outcomes will be measured by a telephone-administered questionnaire two weeks after the initial consultation. A member of the study team will telephone the parent or guardian of each participant fourteen days after they were enrolled into the study. If the researcher is unable to make contact with the parent on this day they will continue attempting to make telephone contact daily for at least three days. If the researcher is unable to make contact after three days of calling, or if the number given has been found to be incorrect or unavailable, the telephone number will be confirmed with the practice which recruited the patient, and checked with directory enquiries. Parents for whom we are still unable to make contact will be sent a brief questionnaire by post. In order to increase the likelihood of response this questionnaire will deal with only the main outcomes – namely re-consultations and use of antibiotics.

The telephone administered questionnaire will ask about consultations with primary, secondary, and out of hours care providers in the two weeks since enrolment, prescriptions for and use of antibiotics, either at the index consultation or subsequently, use of other medications, satisfaction, usefulness of information provided to them in the initial consultation, level of reassurance, intention to consult with similar illnesses in the future, and questions related to the costs of the illness for the family (time off school/work, etc.) An adaptation of the Patient Enablement Instrument [54] will also be completed over the telephone.

#### Follow up data one year after the consultation

Additional measurements will be made one year following the recruitment of the final patient for each practice. Each practice will be sent a list of all recruited patients and asked to provide details of the length of each index consultation, and information on the total number of consultations and the number of consultations for RTI, for the one-year period following each patient's recruitment. To facilitate the collection of data from the practice records the study team will provide practices with support on obtaining consultation length data from their computer system, and beginning and end dates for each one-year follow-up period. General practices will be asked to include information about consultations occurring in other locations (out-of-hours, hospital, out-of-area, etc.) as well as consultations within the general practice.

### Potentially discardable pilot

We conducted a pilot study using two practices in South-East Wales. Both are group practices in areas of high socio-economic deprivation. These practices were randomly assigned by the study statistician, one to intervention and one to control. Recruitment and data collection processes were piloted and participating clinicians provided feedback on these processes and the on-line training. In addition, those clinicians who were in the intervention practice provided feedback on use of the intervention. Minor amendments were made to the on-line training as a result of this experience. As there were no major changes required, data from the pilot practices will be included in the main trial.

### Analysis

Statistical analyses will be conducted using SPSS, STATA, and MLwiN. The primary analysis will be intention to treat and will compare the proportion of patients who re-consulted for the same illness during the fourteen days following recruitment, in the intervention and control arms of the study. A two-level logistic model will be fitted to account for individual and practice-level factors.

Secondary outcomes include: proportion reporting having received a prescription for an antibiotic, proportion reporting having used antibiotics, parental enablement, parental satisfaction, parental assessment of the usefulness of any information received in the consultation, perceived reassurance, and the number of consultations for respiratory tract infections over the following year. These will be analysed using either two-level logistic or linear regression models as appropriate.

We will explore whether a three-level model to control for practitioner factors improves the fit of the model for each analysis.

No formal sub-group analyses are planned. However, exploratory analyses will be conducted of the impact of child's age, presenting symptom complex, and socio-economic background (using postcode of residence) on the effectiveness of the intervention.

### Process evaluation

We will conduct a qualitative process evaluation which will be reported in detail elsewhere. The purpose will be to gain a greater understanding of the clinicians' and parents' perceptions of the intervention (booklet and training) and the elements of it that were perceived to be helpful, and those which were unhelpful. A purposive sample of parents and clinicians in the intervention arm will be invited to participate in semi-structured interviews. These will be conducted between one and four months after enrolling in the study for the parents, and after completion of recruitment for the clinicians.

## Discussion

This will be the first study to explore the impact of using an interactive booklet on respiratory tract infections in children, designed for use within consultations, on re-consultations and antibiotic prescribing. Our intervention has been designed primarily for use with parents, although it could easily be understood by many older children, and acts as an aide-memoir for primary care clinicians. It is applicable to a wide range of childhood respiratory tract infections. The interactive booklet is more likely to be used, understood, and believed, if it is seen to be endorsed by the parent's primary care clinician. Clinicians using the intervention will be provided with training in its use that encourages them to endorse the booklet, to identify and highlight relevant sections of the booklet, and to use it as a prompt to improve communication within the consultation.

The Medical Research Council and others have recommended that complex interventions are based on a theoretical framework [55]. Our intervention is grounded in Social Cognitive Theory and the Theory of Planned Behaviour. Understanding the theoretical underpinnings of the intervention will help us explore which components of the intervention contributed to the effectiveness or lack of effectiveness of the intervention, and will aid in the implementation of the intervention if it is found to be effective [56].

### Outcome measures

Re-consultation for the same illness during the fourteen days following enrolment was chosen as the main study outcome for a number of reasons. Our previous studies have shown nearly one in five children re-consult for the same illness episode [4] Worried parents should not be discouraged from consulting. However, when a significant proportion of parents who have consulted feel the need to re-consult for the same illness, it suggests that they are not being empowered to self-manage these illnesses. Small reductions in resource use in common conditions could result in large savings on a national level. Finally, clinicians are likely to perceive greater pressure to prescribe antibiotics for children who seen for a second or third consultation for the same illness episode.

In addition to other 'clinical' or 'process' outcomes such as antibiotic prescribing and consultations for similar illness over the following year, we will also examine patient-related outcomes including parent reported satisfaction, reassurance, value of information received, and enablement. Enablement is a concept developed by Howie and colleagues which is related to, but different from satisfaction [54] The concept draws on the themes of patient centeredness and empowerment, and on the patient's perceived changes in understanding, coping, and confidence. We adapted the Patient Enablement Instrument for use with parents about care of their children. This involved mainly minor changes to the wording, but did require the item examining impact of the consultation on "ability to cope with life" to be dropped, as this seemed inappropriate when talking about a consultation regarding a third party (the child). No formal validation of this adaptation was conducted. However, its use was found to be acceptable in the pilot.

We will also measure potential adverse effects from the intervention. One UK study randomised 120 parents of infants in a single practice to receive a booklet on childcare followed by a visit from a health visitor, or usual care [57] There was no impact on use of healthcare services, and parents in the intervention arm reported lower levels of feeling confident and knowledgeable than in the control arm. We will measure parental enablement, satisfaction and reassurance, as well as serious adverse outcomes such as complications and hospitalisations.

### Design issues

One central issue relevant to the selection of study design was whether to conduct an efficacy or an effectiveness evaluation. A narrowly defined, closely controlled trial may have allowed us to show an effect that would not be shown in a pragmatic trial. However, such a trial would need to be followed by a further pragmatic trial to show whether the intervention is effective 'in real life general practice'. We have therefore decided to use a pragmatic trial design with broad inclusion criteria. We excluded children under six months of age because symptoms can be more difficult to interpret in very young children, and younger children have higher rates of complications [58]. Children over fourteen years of age were excluded because older children are less likely to consult [3] and be prescribed antibiotics [16]. A disadvantage of a pragmatic approach is an inability to control fidelity of intervention delivery, and this may result in an underestimation of any effect. We will however attempt to gain an understanding of how and whether the intervention was used through the process evaluation.

A further key decision regarding the design of the trial was whether to use an individually or cluster randomised design. Individually randomised trials are generally preferred because cluster trials suffer from a loss of power due to clustering effects. However, since the intervention in this case involves not just the booklet, but a change in the process of the consultation, an individually randomised trial would result in a risk of contamination of intervention delivery. It is not feasible for a clinician who has received training in using the booklet and communicating within the consultation, and has discussed the booklet with some parents, to switch between using this approach and 'usual care' at random.

The use of a cluster design can lead to selection bias at either the level of cluster or the individual. Practices who agree to take part in the study may find that they no longer wish to participate, leading to attrition bias. This would be of greater concern if there is differential drop-out of practices; for example if practices randomised to the control arm are less likely to recruit (resentful demoralisation). We aim to minimise attrition by maintaining regular contact and providing encouragement to all randomised practices, and will monitor and report on the attrition of clusters. Selection bias can also occur in cluster randomised trials where those who are recruiting participants are aware of the allocation given to their cluster [59]. We are not able to blind practices to their allocation as their use of the booklet within the consultation forms part of the intervention. We have attempted to minimise the risk of selection bias by asking practices to recruit sequential eligible patients, and we are trying to measure any selection bias by asking practices to record non-identifiable data on all 'potentially eligible' patients (i.e. all patients who have been given an information sheet about the study, including those who are deemed ineligible, do not consent, or are not recruited for other reasons).

### Other potential sources of bias

Outcomes will be measured primarily through a telephone-administered questionnaire at two weeks. It will not be possible to ensure that the interviewer remains completely blinded to study arm due to the possibility of participants discussing receipt of a booklet during their consultation. However, in order to minimise the risk of information bias, interviewers will not have any information about allocation, and questions will be devised to minimise the chance of participants disclosing which group they are assigned. If a participant discloses their allocation, the interviewer will record this so that these participants can be compared to those in which the interviewer remains blinded to allocation.

Similarly, it was not possible to blind participants to grouping. A 'placebo' booklet was considered. However, use of any booklet is likely to change a consultation, and we wish to assess effectiveness. In order to minimise reporting bias, participants will not be provided with specific information about the intervention or the outcomes being measured. Instead, information sheets state that the study team is interested in determining whether, "the type of information, and the way in which a primary care clinician (GP or practice nurse) communicates this information, can have an effect on your child's illness and the ways in which you deal with it."

### Use of web-based training

A novel aspect of our study will be the use of the Internet to provide training for practices. A clear advantage of this approach is that it precludes the need for a practice visit by the study team, and therefore makes recruitment of practices over a large geographical area possible. In addition, providing training on-line allows clinicians to complete the training at a time and place of their choosing, and at their own pace. Disadvantages of this approach include the time and cost of developing the training; we produced videos, recorded audio-clips, and developed shockwave animated objects for inclusion on the site. Other problems include the loss of face-to-face contact with clinicians, which is likely to have an impact on recruitment, and challenges in measuring training process and outcomes. With regard to the latter, because each clinician is provided with a unique log-in password, we will be able to monitor which pages they access and the amount of time spent accessing the site. This, in addition to written feedback which is requested from all users, will provide us with a measure of amount of training accessed, and their opinions of it.

## Conclusion

This study aims to evaluate the effect of a booklet developed specifically for use within consultations involving children with RTIs, on re-consulting for the same illness episode and antibiotic prescribing. We will determine whether changes in antibiotic use and re-consultation are achieved at the expense of patient satisfaction, and will determine whether the interactive booklet enhances patient enablement. We will evaluate the effect on medicalisation over the subsequent year. If this intervention is found to be effective, even small changes for the commonest acute consultation could have major effects on help seeking behaviour and free up consultations in primary care for other conditions. If not effective, resources spent on developing, printing, and distributing leaflets and booklets on respiratory tract infections in children can be re-directed, and the research agenda can be re-focussed. We believe that this is the first evaluation of the use of written material on respiratory tract infection in children during the consultation.

## Competing interests

The authors declare that they have no competing interests.

## Authors' contributions

NF is the principal investigator and wrote the first draft of the paper. KH helped design the study and is the study statistician. SS helped design the study and manage the trial, FW contributed to the development of the intervention and helped manage the trial, JN helped with data collection and contributed to the trial management, CB conceived of the study, helped design the study, and provided overall leadership to the project. All authors contributed to drafting the manuscript and approved the final manuscript.

## Pre-publication history

The pre-publication history for this paper can be accessed here:


